# Persistent palatal hypertrophy associated with rapid maxillary expansion procedure: Report of a rare case

**DOI:** 10.37796/2211-8039.1007

**Published:** 2020-12-01

**Authors:** Mehmet Melih Ömezli, Damla Torul, Tolunay Avcı

**Affiliations:** Department of Oral and Maxillofacial Surgery, Faculty of Dentistry, Ordu University, Ordu, Turkey

**Keywords:** Complication, food retention, palatal hypertrophy, side effect

## Abstract

Maxillary transversal deficiency (MTD) is a common skeletal problem. If not treated on time, MTD evolve to a more complex malocclusion. This problem can affect facial growth and development. Rapid maxillary expansion (RME) procedure frequently used for the management. This technique is usually successful in young individuals but as the age advances and the articulations of the maxilla with surrounding facial bones get more rigid, it becomes ineffective. Undesirable side effects or results have been reported after use of RME in skeletally mature patients such as buccal tipping of posterior teeth, extrusion, periodontal tissue recession, fenestration of buccal cortex, necrosis of palatal tissue, failure in opening of midpalatal suture, pain, and relapse of expansion. Side effects of RME are often temporary and permanent damages are rarely seen. The aim of this report is to present the management of a permanent side effect of the RME procedure in a 13-year-old child.

## Introduction

Maxillary transversal deficiency (MTD) is a commonly encountered skeletal problem with an incidence of 8.5 to 22% in children and adolescents [1-3]. Syndromes that affect the craniofacial region, mouth breathing, trauma, parafunctional habits like thumb/finger-sucking, and surgical complications of cleft palate repair considered among the factors that contribute to the occurrence of the MTDs [2-4]. Clinically the MTDs usually present with posterior crossbite, crowding of the teeth, high/narrow palatal arch, and mouth breathing [5]. Rapid maxillary expansion (RME) is one of the most commonly used treatment approach to correct MTDs, since the use of fixed palatal expanders in the 1960s [6-9]. During RME treatment to rapidly correct the MTDs heavy forces are transmitted to the maxilla, and as well as the surrounding anatomical structures [10,11]. Based on the magnitude of the force applied, associated with the appliance used, or because of the patient related factors undesirable side effects like pain, swelling, edema, ulceration, gingival recession, root resorption, molar tipping, rotation of the mandible, open bite, and relapse have been reported previously [2,7,12].

The aim of this report is to present the management of palatal mucosal swelling persist after the removal of the RME appliance, and causes food retention in the palatal region.

## Case report

**Fig. 1:**
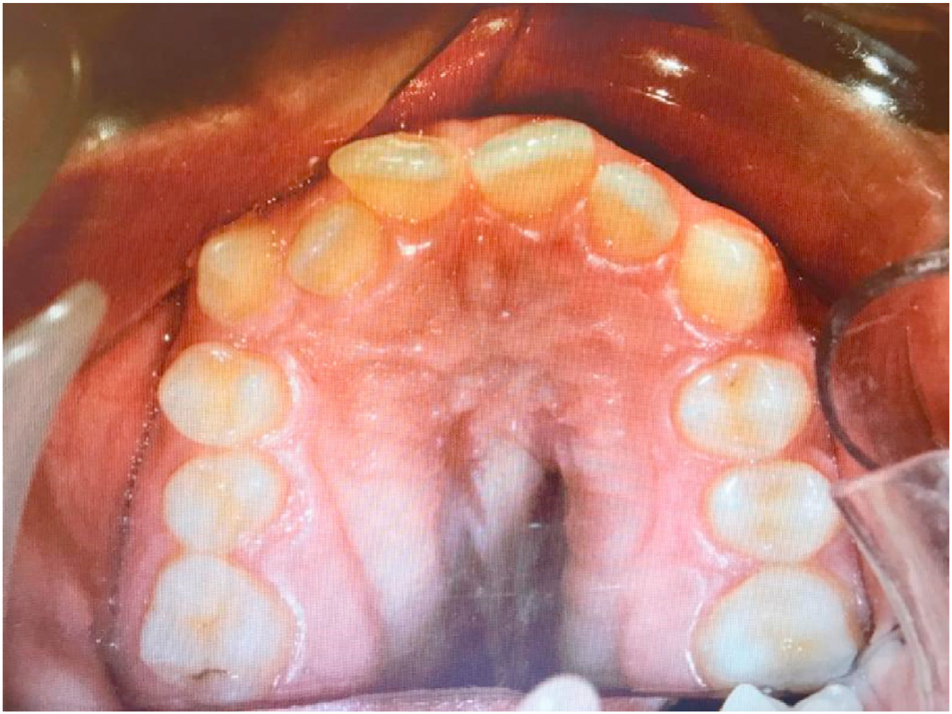
The view of patient's palatal region before orthodontic treatment.

**Fig. 2:**
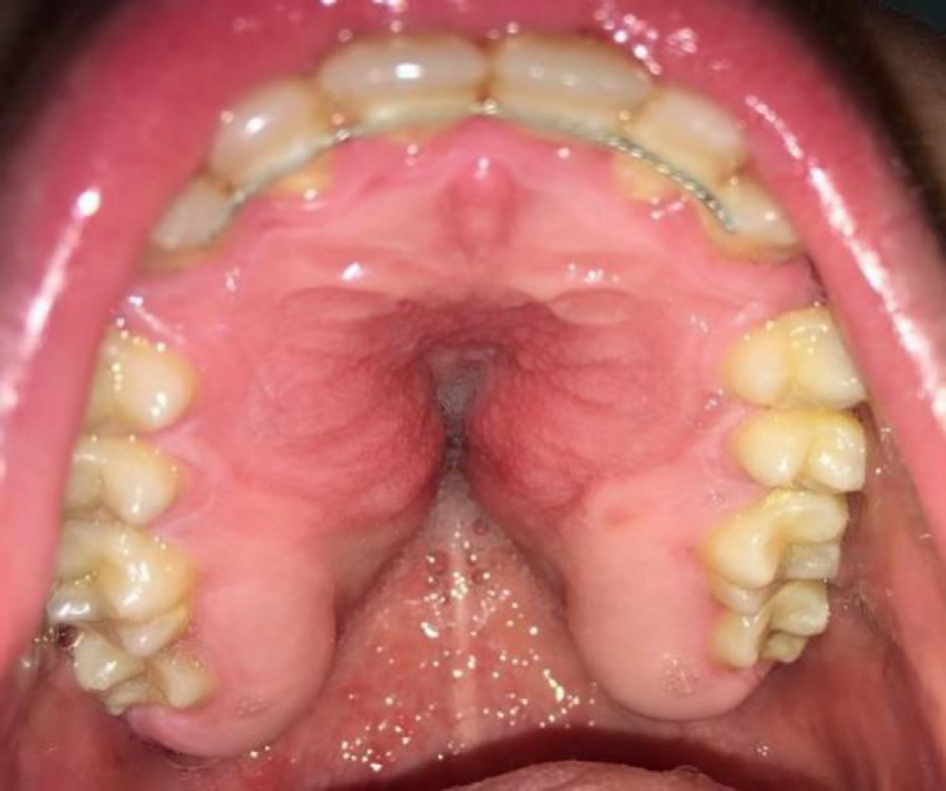
Hypertrophic mucosa on palatal region 3 months after orthodontic treatment.

A 13-year-old male patient was admitted to our clinic with complaints of swelling and food retention in the palatal region. The patient reported that he used RME appliance for 3 weeks and after removal of the appliance, swelling persisted in the palatal region (Figs. 1 and 2). He also added that although the swelling has followed for 3 months, no improvement in the palatal region was observed. Because of the food retention in the palatal region and the inflamed palatal mucosa, surgical correction of this region was planned. Informed consent was obtained from the parents of the patient.

**Fig. 3:**
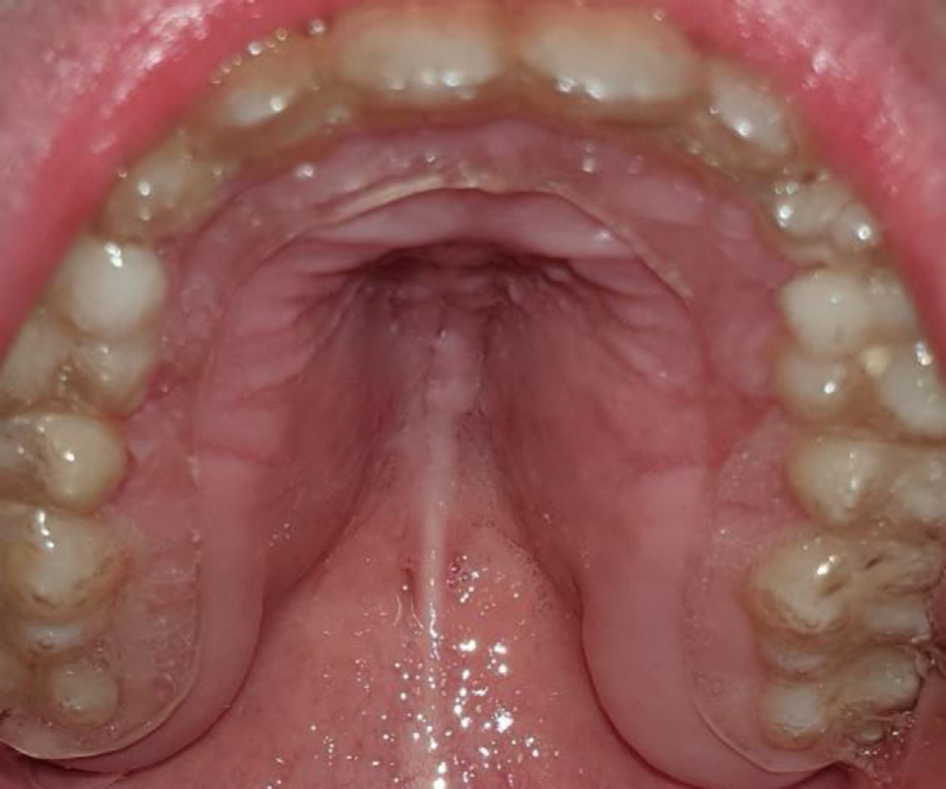
Recovered palatal mucosa 2 months after surgery.

Under general anesthesia, the hypertrophic areas in the palatal region were excised with the help of scalpel and electrocautery. Smooth area that prevents the food retention was created. One week after the operation the healing was non-contributory. In regular follow up visits recovery observed without sequelae (Fig. 3).

## Discussion

RME procedure is widely preferred treatment option for the management of MTDs [7,13]. Undesirable side effects like decubitae or swelling in palatal mucosa, pain, tooth tilting, root resorption, caries and periodontal problems have been reported after the RME procedure [2,7,14]. Schuster et al. suggested that side effects of RME are often temporary and permanent damages rather rare [14]. It is reported that the side effects of RME tend to be greater in adeloscence than it is in children associated with the degree of skeletal maturity [13,15,16].

Capelozza et al. reported to observed side effects such as pain, edema, and ulceration frequently after RME in mature patients [12]. In the present case although at 13 years of age, a permanent swelling due to the mucosal hypertrophy in the palatal region was observed. Alterations in palatal mucosa may be seen during RME treatment. However, palatal mucosa irritations reported healing completely within a short period of time once the appliance is removed [14]. Also, Brunetto et al. reported that the anatomy of the palatal region may affect the success of the RME procedure [17]. In the present case the patient has a high-arched atypical palatal anatomy before the initiation of the treatment, and during RME procedure swelling was occurred in the palatal mucosa that causes food retention and prevents maintenance of oral hygiene. We think that the anatomy of the palatal region increases the susceptibility to the occurrence of this complication by preventing the application of force homogeneously. In addition, we think that uncontrolled use of force triggers permanent hypertrophy in the palatal mucosa. Subsequently, increased hypertrophy caused food retention and ultimately led to inflammation of the mucosa.

## Conclusion

Although rare, permanent complications may occur in immature patients after RME treatment. Therefore, to prevent these complications appropriate treatment approach/appliance should be chosen, and regular follow-up with a strict oral hygiene regimen should be provided. It should not be forgotten that in many cases, some complications can be prevented by switching to a different appliance or by interrupting its use.
